# Alteration of interleaflet coupling due to compounds displaying rapid translocation in lipid membranes

**DOI:** 10.1038/srep32934

**Published:** 2016-09-06

**Authors:** Ramon Reigada

**Affiliations:** 1 Department de Química Física and Institut de Química Teòrica i Computacional (IQTCUB), Universitat de Barcelona, c/ Martí i Franqués 1, Pta 4, 08028 Barcelona Spain

## Abstract

The spatial coincidence of lipid domains at both layers of the cell membrane is expected to play an important role in many cellular functions. Competition between the surface interleaflet tension and a line hydrophobic mismatch penalty are conjectured to determine the transversal behavior of laterally heterogeneous lipid membranes. Here, by a combination of molecular dynamics simulations, a continuum field theory and kinetic equations, I demonstrate that the presence of small, rapidly translocating molecules residing in the lipid bilayer may alter its transversal behavior by favoring the spatial coincidence of similar lipid phases.

Cell membranes are heterogeneous lipid bilayers^1^ whose internal organization has been investigated from different perspectives. The most common corresponds to the lateral (in-plane) lipid organization. It has been proposed that the cell membrane is structured in liquid-ordered (Lo) cholesterol-rich nanodomains (also called lipid rafts^2^) surrounded by a liquid-disordered (Ld) lipid phase. Lo/Ld phase segregation is macroscopically observed in ternary lipid membrane mixtures containing cholesterol^3^,^4^. Although controversial^5^,^6^, the idea of “lipid raft” nanodomains as a basic organizing principle has been useful to understand the connection between cell membrane structure and functionality^7^. While there have been many studies devoted to the in-plane membrane configuration^8^, it is only recently that attention has been turned to its transversal organization^9^,^10^, and in particular, to the extent of correlation of lipid domains in the two leaflets of the bilayer. This issue is important to understand many relevant biological functions taking place in the cell membrane, for example, the mechanism by which proteins on both sides of the membrane co-localize during signaling events^11^, the transversal spatial coincidence of specific lipid phases required in immunological responses^12^, the activity of particular ion channels^13^ or inserted transmembrane proteins^14^,^15^,^16^, etc.

Coupled phase-segregating bilayer leaflets have been typically studied using phenomenological free energies^9^,^17^,^18^,^19^,^20^. In short, these approaches suggest the competition between two interleaflet coupling effects. First, lipid tail ordering interactions across the bilayer midplane^17^,^18^,^20^ is responsible for a surface interleaflet tension that promotes the transversal alignment (registration) of similar lipid phases. Second, a line tension that comes out from the hydrophobic lipid tail mismatch between the leaflets, favoring a uniform bilayer thickness and therefore non-symmetric membranes (antiregistration). Phase registration and antiregistration modes are schematically presented in Fig. 1. The two competing tensions have a different impact depending on the size of the lipid domains: since the phase symmetry is promoted by surface tension (energy per surface unit) oppositely to the line tension (energy per length), as domain area increases, larger domains are expected to be always in registration. This explains why domain registration is usually observed in microscopy experiments^21^. In the biological context, however, membrane lipid domains are presumed to develop at the nanoscale. The small size of membrane domains *in vivo* could be due to curvature effects^22^,^23^, critical fluctuations^24^, active recycling^25^,^26^ or other mechanisms that are still being debated. In any case, it is worth noticing that at such short scales, both surface and line tension penalties may be of the same order, and therefore both registration and antiregistration behaviors could take place^19^.

Despite the simplicity of the argument based on the balance between domain surface and edge tensions, it currently remains undetermined exactly how leaflets of a bilayer are coupled together, and whether cell membranes display registration or antiregistration of their lipid domains. This naturally brings up the role of the specificity of the lipids forming the membrane and their particular chemistry on the interaction between leaflets in a bilayer. At this point, molecular dynamics (MD) offers a powerful technique to address this issue at the molecular level. Recent atomistic MD simulations^27^ have been conducted for asymmetric ternary bilayers comprising an unsaturated lipid, a saturated lipid and cholesterol. In these simulations one layer was placed in the Lo/Ld coexistence region whereas the composition of the other was progressively modified across the first order phase boundary. Despite the limitations of atomistic MD to achieve the time- and length-scales required to capture membrane domain formation, bilayer registry was found and, remarkably, an incipient phase segregation induction effect between layers was also observed^27^. The use of coarse-grained (CG) MD allows the simulation of larger membrane patches at longer times so that both in-plane and transversal membrane organization can be fully analyzed. In particular, Perlmutter & Sachs^28^ found an intriguing connection between lipid chain-length and the registration/antiregistration behavior in phase segregating membranes. They run a series of CG MD simulations in which the length of the saturated lipid was varied, and observed that Lo domains containing lipids with the shorter tail were in register whereas those containing lipids with longer chains were antiregistered. The connection with the former energetic arguments is clear: long saturated lipid chain results in a larger thickness mismatch between Lo and Ld segregating domains, thus favoring the antiregistration behavior^28^. Moreover, they also analyzed how compositional asymmetry was coupled to phase segregation and the structure of the individual leaflets, providing a detailed picture of the mechanisms of interleaflet coupling.

The effects on the transversal membrane organization caused by non-lipid compounds that may be eventually added to a bilayer system have been also analyzed by means of CG MD simulations. In particular, the addition of chloroform (chlf) molecules to antiregistered phase segregating bilayers turned out to result in phase registration after a few microseconds^29^. This effect is interpreted in terms of an entropic gain that would favor frequent chloroform commuting between the two leaflets, and can be extended to other small mildly polar molecules with the ability to rapidly translocate between the layers of a bilayer.

In this paper I focus on the effect of rapidly translocating compounds on the transversal domain symmetry of phase segregating membranes. The construction of a theoretical model for this effect and its corresponding analysis provide a more complete picture of the transmembrane organization of lipid domains *in vivo* under the influence of particular compounds (drugs, anesthetics, etc.) with possible relevance to signaling and the anesthetic action. First, I summarize the main results from CG MD simulations of compositionally symmetric ternary bilayers where the mentioned effect is clearly observed for nano-scale domains that may resemble raft structures *in vivo*. Second, the continuum field model based on a free energy formalism for the two-leaflet segregating bilayer is proposed by considering the contributions due to the presence of the non-lipid compound that is assumed to preferentially reside in one particular lipid phase. At this point, a simple analysis already anticipates the effect of the non-lipid compound in favor of membrane domain registration if this compound displays a fast translocation rate between leaflets. Third, the corresponding kinetic equations are derived and investigated by means of their linear stability analysis. A sort of kinetic stability diagram is obtained and the conditions for the kinetic stabilization of registered modes are determined. Finally, nonlinear simulations of registered and antiregistered emerging membrane phases are performed, demonstrating how such structures develop through the instability of the initial uniform state.

## Results and Discussion

### Coarse-grained molecular dynamics simulations

CG MD simulations have been conducted for two different types of phosphatidylcholine (PC) membrane systems comprising an unsaturated lipid (DUPC, a PC with two double-unsaturated 16:2 oleoyl tails), a saturated lipid (either DLPC with two saturated 12:0 lauroyl tails or DSPC with two saturated 18:0 stearoyl tails) and cholesterol (chol). Bilayers were composed of 828 DUPC molecules (42.6 mol%), 540 saturated PC lipids (27.8 mol%) and 576 chol molecules (29.6 mol%), randomly and equally distributed in the two leaflets. Membrane systems have been conveniently hydrated with 12600 water beads and simulated for several microseconds. Other simulations details and protocols are summarized in Methods.

**Figure 2:**
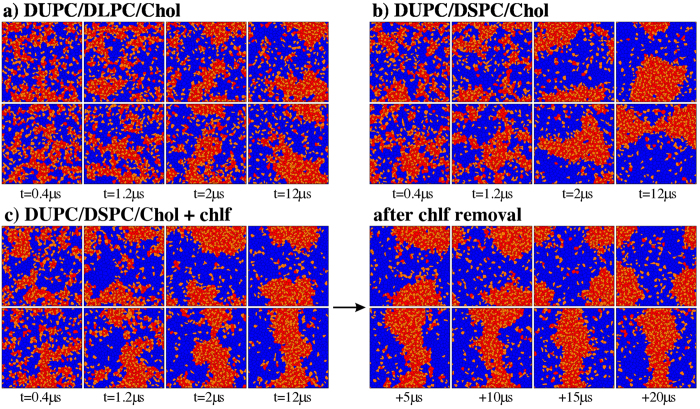
Summary of molecular dynamics simulations. Sequence of phase segregation for different MD simulations. Voronoi polygons are filled with a colour corresponding to each lipid component: unsaturated lipid (DUPC in blue), saturated lipid (red) and cholesterol (orange). Upper and lower rows correspond to each membrane leaflet. (**a**) Ternary system with a short-tailed saturated lipid (DLPC) resulting in domain registration. (**b**) Ternary system with a long-tailed saturated lipid (DSPC) resulting in domain antiregistration. (**c**) Inclusion of chloroform to the DUPC/DSPC/Chol system leads to a registration of segregating domains, whereas chloroform removal recovers the antiregistration behavior.

Temporal evolution of both simulated membrane systems display phase separation: the saturated lipid form a packed lipid phase (Lo) together with cholesterol, segregated from a disordered phase rich in DUPC (Ld). The proportion of lateral neighboring pairs of the same lipid phase (see Methods) after 12 μs of simulation exceeds the 85%, indicating clear phase segregation. A sequence of the segregation process is shown in Fig. 2a,b for the two layers of the DUPC/DLPC/chol and DUPC/DSPC/chol membranes, respectively. Each segregating phase exhibit distinct structural and dynamical characteristics. The Lo phase is richer in cholesterol (42.2 and 44.0 mol% for the membranes with DLPC and DSPC, respectively) than the Ld phase (17.1 and 15.3 mol% for the membranes with DLPC and DSPC, respectively), and it forms thicker layers than the Ld phase (see below). Lateral mobility of PCs in the ordered phase is reduced (5.1 ± 0.4 and 4.3 ± 0.4 10^−8^ cm^2^s^−1^ for the membranes with DLPC and DSPC, respectively) respect to mobility in the liquid disordered region (8.8 ± 0.4 and 8.1 ± 0.4 10^−8^ cm^2^s^−1^ for the membranes with DLPC and DSPC, respectively), in agreement with experimental observations^30^.

Importantly, the membrane containing the longest-tail saturated lipid (DSPC) shows a clear antiregistration behavior, whereas the membrane containing the shortest saturated lipid tails (DLPC) presents an alignment of the segregating domains (registration). Quantification in terms of area registration percentage (see Methods) after 12 μs of simulation yields to a 66% of phase coincidence for the membrane with the short saturated lipid DLPC whereas this percentage is reduced to 32,5% for the bilayer with DSPC. These results agree with those already reported in Refs 28 and 29, and they can be easily explained by the competition between surface and line interdomain tensions. A piece of membrane with the two leaflets in a Lo phase made of DSPC and chol molecules is significantly thicker (4.7 nm) that the Ld phase composed by the coincidence of two domains of DUPC lipids (3.8 nm). Such thickness mismatch plays against transmembrane colocalization of equal phases and promotes phase asymmetry in the DUPC/DSPC/chol system (Fig. 2b). Instead, the thickness of a Lo membrane patch composed by DLPC and chol molecules is rather similar to the DUPC Ld membrane patch, so phase alignment is favored in this case (Fig. 2a).

The simulation for the DUPC/DSPC/chol bilayer system has been repeated in the presence of 1500 chlf molecules (0.77 chlf molecules per lipid, below the saturation condition of 3–4 molecules per lipid^31^). Chloroform molecules were initially added to the membrane system in the aqueous phase and became almost totally absorbed by the bilayer after a few nanoseconds. Visual inspection of temporal sequence for this chlf-containing bilayer system plotted in the left panels of Fig. 2c reveals the powerful influence of chloroform to promote phase registration in contrast to the same membrane in the absence of this compound. Addition of chlf dramatically increases the area registration percentage to a 65%, converting a clear antiregistration behavior into a symmetric transversal configuration^29^. I have also checked that the reported effect is robust and independent of the initial bilayer configuration. As an example, depletion of chlf molecules from the final snapshot of the DUPC/DSPC/chol + 0.77chlf/lipid phase-aligned membrane system results in transversal antiregistration after a few microseconds (see the temporal sequence in the right panels of Fig. 2c).

Negative control simulations have been conducted for the DUPC/DSPC/chol membrane by adding 1500 molecules of two compounds with distinct polarity: carbon tetrachloride (with null polar affinity) and ethanol (with a larger polar character than chlf). Simulations show that neither of the two compounds causes the synchronization effect produced by chloroform. Addition of carbon tetrachloride slightly increases the area registration proportion only to a 39%, whereas ethanol does not alter transversal synchronization at all.

**Figure 3:**
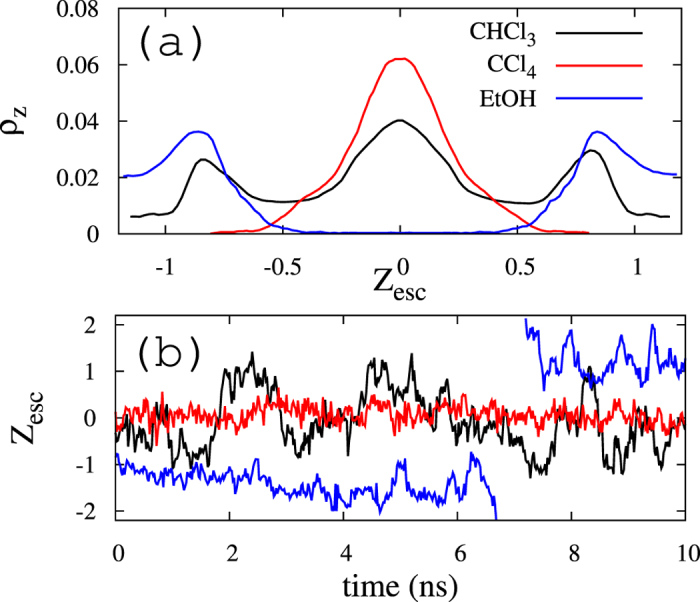
Transversal behavior of added compounds. (**a**) Chloroform, carbon tetrachloride and ethanol density distribution probabilities in the z-axis (normal to membrane surface), ρ_z_. The profiles are plotted in a scaled distance with respect to the bilayer center, Z_esc_, where the position of the opposite phosphate beads are fixed to Z_esc_ = ±1. (**b**) Representative trajectories of chloroform, carbon tetrachloride and ethanol molecules tracked for a 10 ns period of time in the DUPC/DSPC/Chol membrane system.

The alignment effect can be studied at the molecular detail by analyzing the behavior of chloroform molecules in the simulated bilayer. Two aspects of their distribution are important. In respect of the in-plane behavior, chlf displays a clear preference to occupy the more disordered (Ld) regions of the phase-segregated membrane. In the reported DUPC/DSPC/chol membrane the fraction of chlf molecules that are proximal to the DUPC lipids (Ld phase) is approximately a 83% larger than the corresponding to a random distribution, whereas DSPC and chol (Lo phase) show a clear depletion of chloroform (see ref. 24 for more details). Such preference was also confirmed by atomistic MD simulations^32^,^33^. In respect of the transversal behavior, the time averaged (over the last 4 μs of simulation) density profile for chlf molecules is computed for the direction normal to the membrane surface and plotted in Fig. 3a. Notice that chloroform spans the hydrophobic region of the membrane with a preference for the inner region of the lipid/water interface, just below the phosphate groups^32^,^33^. Moreover, the inspection of chlf trajectories inside the bilayer reveals that chloroform molecules display a very dynamic behavior that combines short confinement periods close to the interface with fast jumps from one leaflet to the other. According to atomistic^32^,^33^ and CG^29^ simulations chloroform leaflet translocation frequencies are of the order of a few fractions of ns^−1^. A typical transversal trajectory of a chlf molecule in the simulated DUPC/DSPC/chol + 1500chlf system is plotted in Fig. 3b confirming the nanosecond scale for chloroform translocation.

Summing up the in-plane and the transversal behavior of chloroform in phase segregated bilayers, the mechanism by which this compound promotes phase symmetry can be attributed to a entropic effect. Transversal coincidence (registration) of disordered domains where chlf molecules are preferentially placed favors their fast transversal dynamics and avoids their confinement in one leaflet. It is important to notice that this effect can be shared with other compounds that display both mentioned aspects: a preference for one of the two segregating lipid phases and a fast transversal dynamics inside the bilayer. The molecular characteristics of chlf that gives rise to its coupling effect are, therefore, its moderate polar affinity that causes this compound to partition inside the bilayer with a preference to interact with the lipid/water interface, and its small size that allows fast translocation form one leaflet to the other.

Negative controls performed with carbon tetrachloride and ethanol confirm the entropic origin of the reported effect: compounds displaying fast translocation between layers promote transversal domain coincidence in order to avoid its confinement in one of the two membrane leaflets, whereas compounds lacking in this degree of freedom do not cause interleaflet synchronization. Carbon tetrachloride molecules, without polar affinity, are preferentially located at the more inner hydrophobic region of the membrane (see density profile in Fig. 3a), and they perform trajectories around the bilayer center that are more confined than those displayed by chlf molecules (Fig. 3b). As a consequence, only a tiny interleaflet coupling effect is observed. Ethanol molecules are prone to occupy the lipid/water interfaces but avoiding the inner hydrophobic bilayer core (see Fig. 3a). Since ethanol molecules rarely traverse the hydrophobic section of the membrane (see Fig. 3b), no synchronizing effect is found.

### Continuum field theory: membrane free energy

To provide a more general framework for the reported synchronizing effect, a theoretical model for a lipid membrane has been attempted by taking into account the influence of a generic rapidly translocating compound (hereafter referred as non-lipid or added compound). The phenomenological modeling of a membrane is usually based on a simplified description of its microscopic degrees of freedom by means of a minimum number of order parameters. The energetics of the system is then expressed in terms of these variables. As starting point I adopt the typical free energy description of a multicomponent lipid membrane consisting in two coupled monolayers. For simplicity, nearly flat bilayers are considered so that elastic curvature terms will not be included in the energetic description. The lipid mixture at each layer is considered as a pseudo-binary system characterized by the order parameters *ϕ*_1_(*r*) and *ϕ*_2_(*r*) for each one of the bilayer leaflets. As suggested in many continuum approaches describing Lo/Ld phase segregation^17^,^18^,^20^, the free energy contribution due to the lateral interaction between the different components at each monolayer can be expressed (in k_B_T units) as a Landau expansion^34^:


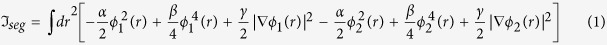


where *β* is a positive coefficient and the sign of the parameter α determines the thermodynamic state of the lipid mixture. For a positive value of *α*, equation (1) captures phase segregation of two phases that may correspond (arbitrarily) to the Lo and Ld lipid phases: *ϕ*^(*Lo*)^ = +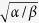 and *ϕ*^(*Ld*)^ = −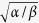. The parameter *γ* accounts for the line domain tension in a monolayer. The two leaflets interact via a direct surface interleaflet tension^17^,^18^,^19^,^20^


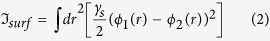


where *γ*_*s*_ is the surface tension coefficient, and an indirect line tension penalty due to the energy cost associated to membrane thickness mismatch^17^,^18^,^19^,^20^. The latter contribution can be expressed as proportional to the square of the local thickness variation. Assuming a proportional relation between each layer thickness and its corresponding lipid ordering parameter, this energy penalty can be straightforwardly written as


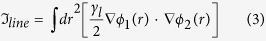


where *γ*_*l*_ is the thickness mismatch line tension coefficient. The effect of the non-lipid compound can be included in the free energy description in the simplest form by adding the following energy contribution


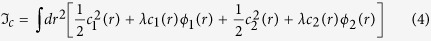


where *c*_1_(*r*) and *c*_2_(*r*) correspond to the local concentration deviation of the non-lipid compound at each leaflet respect to its average membrane concentration, and *λ* is a positive affinity parameter that accounts for the preference of the added compound for the Ld lipid phase.

### Free energy analysis

For a phase segregating mixture (*α* > 0) the energetics of the registration and antiregistration modes at different wave numbers can be easily compared by a simple analysis. Equations (1, 2, 3, 4) are applied to one-dimensional modulated periodic phases with wave number *q* and different shift phase angles. In particular, I choose *ϕ*_1_ = *ϕ*_*m*_ cos (*qx*), *ϕ*_1_ = *ϕ*_*m*_ cos (*qx* + *ϕ*), *c*_1_ = *c*_*m*_ cos (*qx* + *ϕ*_1_) and *c*_2_ = *c*_*m*_ cos (*qx* + *φ*_1_ + *φ*_c_, where *ϕ*_*m*_ and *c*_*m*_ are the amplitudes, *ϕ* is the phase shift between the lipid compositional order parameters in the two leaflets, *ϕ*_1_ is the phase shift between the lipid order parameter and the non-lipid concentration in leaflet 1, and *φ*_*c*_ is the phase shift between the non-lipid concentration parameters in the two layers. The total free energy for a given wave number can be obtained:


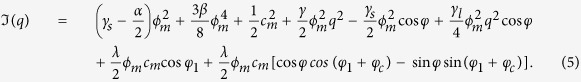


In the absence of the effect caused by the non-lipid compound (*λ* = 0), the balance between the interfacial and thickness line tension contributions (fifth and sixth terms in equation (5), respectively) determines the bilayer transversal behavior. Such balance reveals that small domains (large *q*) are stabilized in antiregistration (*φ* = *π*), whereas large ones are less penalized when aligned (*φ* = 0). The critical wave number *q*_*c*_ is obtained by applying the condition ℑ(*q*_*c*_, *φ* = 0, *λ* = 0) = ℑ(*q*_*c*_, *φ* = *π*, *λ* = 0), resulting in


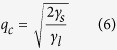


From molecular dynamics simulations^17^,^35^ *γ*_*s*_ ≈ 0.1 k_B_T/nm^2^ was estimated. The thickness line tension parameter was experimentally measured in a phase segregating lipid vesicle^36^ as *γ*_*l*_ ≈ 0.25 k_B_T/nm. According to equation (6), domains with a radius larger than 2*π*/*q*_*c*_ ≈ 3.5 nm might show phase registration. The latter value of *γ*_*l*_ corresponds to a layer height mismatch smaller than 3 Å. The dependence of line tension on height mismatch can be estimated by a simple molecular model^37^, and predicts values up to 3–4 times larger for layer mismatches of the order of those found in our DUPC/DSPC/chol simulated membrane (≈4, 5 nm). In this case, the predicted critical domain radius increases up to 7 nm.

The effect due to the non-lipid compound on the registration/antiregistration balance requires the analysis of all terms in equation (5) and the additional optimization of the two phase shifts *φ*_1_ and *φ*_*c*_. Two extreme situations are analyzed. First, I consider the scenario of a non-translocating compound, so that its concentrations at the two leaflets are not necessarily correlated. A positive value of *λ* implies a preference of the non-lipid compound to be placed in the disordered phase, *ϕ*^(*Ld*)^ < 0, and in this case this happens in both leaflets. As a consequence, the minimum energy is achieved for *φ*_1_ = *π* and *φ*_*c*_ = *φ*, and the same conclusions for the optimum lipid phase shift *φ* are obtained than in the absence of the non-lipid compound. Therefore, when the added non-lipid compound does not translocate between leaflets, the registration/antiregistration balance of lipid phases is not altered and equation (6) is still valid. For rapidly translocating compounds the situation is, however, completely different. In this case, the distribution of non-lipid molecules is assumed to be symmetric respect to leaflet exchange (i.e.; *φ*_*c*_ = 0) and this, in combination with higher non-lipid concentration in the disordered domains (*φ*_1_ = *π*), energetically favors phase registration: *φ* = 0, (see last two terms in equation (5)). By applying ℑ(*q*_*c*_, *φ* = 0, *φ*_*c*_ = 0) = ℑ(*q*_*c*_, *φ* = *π*, *φ*_*c*_ = 0) the modified critical wave number is obtained


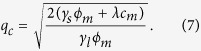


Notice that transversal spatial coincidence of the non-lipid compound acts as an additional surface tension that shifts the lipid registration behavior to larger wave numbers; namely to smaller domains (compare equations (6) and (7)). Such simple analysis points out the main conclusion of this Paper: addition of a compound displaying preference for a particular lipid environment and a fast exchange rate between the layers of the membrane favors phase registration of segregating domains.

### Kinetic equations and linear stability analysis

The temporal evolution of the membrane lipid composition order parameters can be described by means of a conserved scheme,


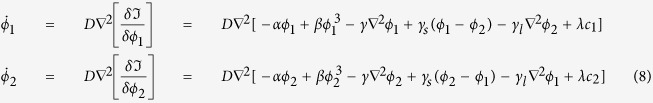


where D stands for lipid diffusivity and is fixed to unity in model units. According to the Cahn-Hilliard theory^38^ 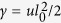 where the interaction energy is about *u* ≈ 1 k_B_T and the characteristic lateral interface width between domains can be estimated as *l*_0_ ≈ 2 nm. By choosing *γ* = 1, the length unit in our equations becomes fixed to *l*_*u*_ = 2 nm. As anticipated^24^,^35^,^36^,^37^, *γ*_*s*_ ≈ 0.1 k_B_T/nm^2^ and *γ*_*l*_ ≈ 0.25–0.75 k_B_T/nm, that correspond to *γ*_*s*_ ≈ 0.4 and *γ*_*l*_ ≈ 0.5–1.5 in model units, respectively. Lateral diffusion of most lipids are of the order of *D* = 10 μm^2^/s^29^,^39^, so that by using 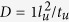 the time unit in our model equations is automatically fixed to *t*_*u*_ = 4.10^−7^ s.

The kinetic behavior of the distribution of the added compound at each leaflet also follows a conserved scheme for the lateral (in-plane) transport supplemented with terms taking into account the exchange between leaflets


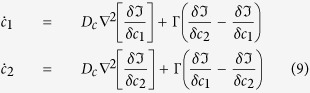


where Γ is a transversal mobility parameter and D_c_ stands for lateral diffusivity. As it was done for the free energy analysis, the case of a fast translocating compound is analyzed. In this scenario, Γ → ∞, equilibrium is reached when the chemical potentials of the added compound at both leaflets, (*δ*ℑ)/(*δc*_1_) and (*δ*ℑ)/(*δc*_2_), are locally equal; namely when *c*_1_(*r*) = *c*_2_(*r*). The kinetics of the fast translocating compound can be therefore described at both layers of the membrane by a single concentration parameter *c(r*) = *c*_1_(*r*) = *c*_2_(*r*), and its evolution follows


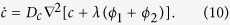


The linear stability analysis of the stationary homogeneous solutions 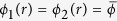 and 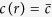 with respect to small perturbations *δϕ*_1_, *δϕ*_2_, 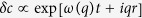 can be obtained straightforwardly from the linearized equations and the corresponding 3 × 3 linearization matrix:


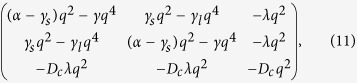


where, for simplicity, symmetrical overall leaflet compositions 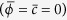 have been assumed.

In the absence of a fast translocating compound (unperturbed membrane), the kinetics of the different modes can be typically described by the upper-left corner 2 × 2 matrix (11). The growth rates *ω(q*) correspond to the largest eigenvalues of the associated Jacobian that are given by


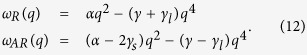


*ω*_*R*_(*q*) corresponds to registered domain formation modes (thickness mismatch penalty is added to the monolayer domain line tension) whereas *ω*_*AR*_(*q*) describes the growth kinetics of antiregistered perturbations (large *γ*_*s*_ and low *γ*_*l*_ destabilize these modes). For deeply phase-segregating case and moderate interleaflet coupling (*α* > 2*γ*_*s*_, *γ* > *γ*_*l*_) small wave numbers *q* are always stable for both solutions and become unstable above certain limits: *ω*_*R*_(*q*) > 0 for *q* ∈ 
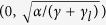
 and *ω*_−_(*q*) > 0 for *q* ∈ 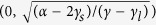. Although both segregation modes are stable at long wavelengths, the registered modes grow quicker than the antiregistered ones at sufficiently small *q* < *q*_0_, where *ω*_*R*_(*q*_0_) = *ω*_*AR*_(*q*_0_). As anticipated by free energy arguments, as soon as the domains grow above a length 
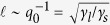
, the surface interleaflet penalty dominates over the line thickness mismatch tension and registered patterns are predicted.

The balance between the two interleaflet couplings results in competing registration and antiregistration modes at different length scales. When a bilayer starts to phase separate any spinodal instability compete to determine the dominant initial demixing mode. If the registration mode is the fastest growing, the equilibrium coexistence of phase-aligned domains is directly accessed. However, if the antiregistered mode is the fastest growing, spinodal decomposition to nonaligned domains occurs first, leading to local asymmetry throughout the membrane. Despite the thermodynamic preference for transversally coincident phases at long wavelengths, such anticorrelated domains can be kinetically preferred so that before equilibrating to a registered configuration the bilayer must escape a metastable locally asymmetric state. Such process has been analyzed in detail by Williamson & Olmsted^19^,^20^ by means of a free energy formalism based on microscopic interactions for the two interleaflet coupling contributions and supported by Monte Carlo simulations. They found that when the initial demixing steps were dominated by the antiregistered mode the bilayer either reached the registered configuration by nucleation out of the antiregistered state or remained metastably trapped in antiregistration^19^,^20^.

**Figure 4:**
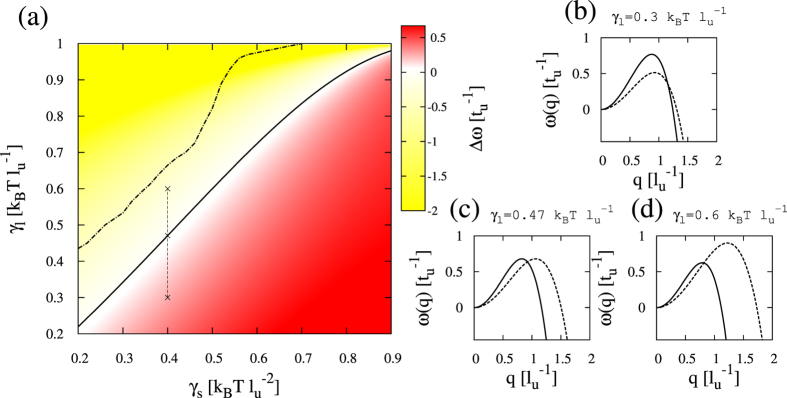
Kinetic diagram and growth rate behavior in unperturbed membranes. (**a**) Kinetic stability diagram, Δ*ω*, for the pure lipid system (*D*_*c*_ = 0, *λ* = 0) and varying the interleaflet surface tension *γ*_*s*_ and the thickness mismatch tension *γ*_*l*_. The other model parameters are fixed to *α* = 2, *γ* = 1, and 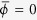. Yellow (red) indicates the parameter region where antiregistration (registration) modes dominate the initial demixing stages. The black curve corresponds to the analytical prediction for 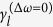 in equation (13). The dot-dashed curve corresponds to the boundary between registered and antiregistered regions directly computed from numerical simulations, 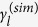. (**b**–**d**) Illustrative growth rates *ω*_*R*_(*q*) (solid) and *ω*_*AR*_(*q*) (dashed) corresponding to the kinetic diagram in panel (**a**) for *γ*_*s*_ = 0.4 and different values of *γ*_*l*_ traversing the kinetic boundary (see vertical dashed line in panel (**a**)).

Each mode exhibits a maximum growth rate, 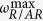, and the difference between them, 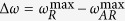, determines which mode dominates the initial segregation process once the bilayer is quenched. The representation of Δ*ω* as a function of different model parameters provides a sort of “kinetic” stability phase diagram that characterizes the competition between the two segregating modes. Figure 4 displays the stability diagram for varying the two interleaflet coupling parameters, *γ*_*s*_ and *γ*_*l*_, supplemented with some examples for the growth rates *ω*_*R*_(*q*) and *ω*_*AR*_(*q*) at the different regions of the kinetic diagram. Registration and antiregistration regions are separated by a boundary that can be obtained analytically from equations (12) by applying the condition Δ*ω* = 0, resulting in


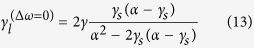


Notice that the linear stability analysis does not provide any information about the transition from a metastable anticorrelated state found in the Δ*ω* < 0 kinetic diagram region to the registered equilibrium configuration. Nonlinear numerical simulations of the kinetic equations should be performed to analyze such issue in detail (see Numerical Simulations). An exhaustive investigation based on Monte Carlo simulations was reported in Refs 19 and 20.

The effect on the registration/antiregistration balance reported so far due to a fast translocating non-lipid compound requires the full resolution of the 3 × 3 linearization matrix (11). Three growth rates are then obtained: the same rate for antiregistered modes *ω*_*AR*_(*q*) obtained from the pure lipid system in equation (12) that remains unaltered, a growth function for damped modes *ω(q*) < 0, and a modified growth rate for registered modes


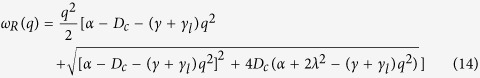


**Figure 5:**
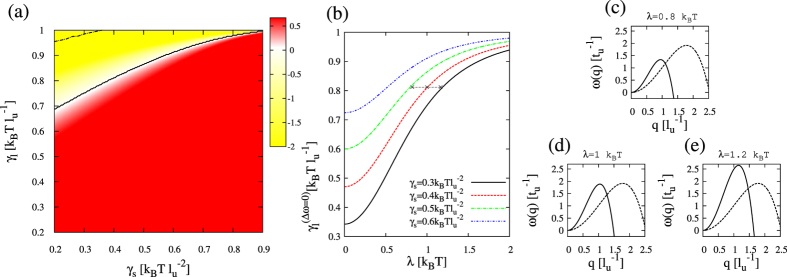
Kinetic diagram and growth rate behavior in the presence of a fast translocating compound. (**a**) Kinetic stability (*γ*_*s*_, *γ*_*l*_) diagram for a segregating membrane in the presence of a rapidly translocating compound (*D*_*c*_ = 10, *λ* = 1). The other model parameters are fixed to *α* = 2, *γ* = 1, 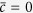 and 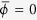. Yellow (red) indicates the parameter region where the antiregistration (registration) modes dominate the initial demixing stages. The solid curve stands for 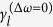 whereas the dot-dashed curve corresponds to the boundary between registered and antiregistered regimes directly computed from numerical simulations, 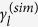 (more details in Fig. 10). (**b**) Dependence of 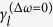 with the coefficient *λ* at different values of *γ*_*s*_. (**c**–**e**) Illustrative growth rates *ω*_*R*_(*q*) (solid) and *ω*_*AR*_(*q*) (dashed) corresponding to panel (**b**) for *γ*_*s*_ = 0.4, 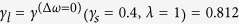 and different values of *λ* traversing the kinetic boundary (see horizontal dashed line in panel (b)).

The non-lipid compound is assumed to be much smaller than any lipid membrane component and therefore to display a much larger lateral mobility in the membrane than the lipid components. For example, chloroform exhibits a lateral diffusion coefficient in the range 30–600 μm^2^/s according to CG MD^29^, so here I set *D*_*c*_ = 10 in model units. The preference of the added compound to occupy the disordered lipid phase^29^,^32^,^33^ justifies a choice for the parameter *λ* of the order of k_B_T. As shown in Fig. 5a, the addition of a rapidly translocating compound expands the area corresponding to the registered regime in the kinetic diagram. The boundary value for the thickness line tension coefficient 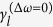 has been numerically computed, displaying larger values than the prediction in equation (12) and an increasing behavior with the affinity parameter *λ* (Fig. 5b). As it is shown in Fig. 5c–e, the mode *ω*_*R*_(*q*) is stabilized, and as a consequence the registered behavior is kinetically preferred at larger values of *γ*_*l*_ than in the unperturbed membrane case. The agreement between the energetic prediction reported in the previous section and the modification of the kinetic preference in favor of the registered mode is therefore clear.

### Numerical simulations

**Figure 6:**
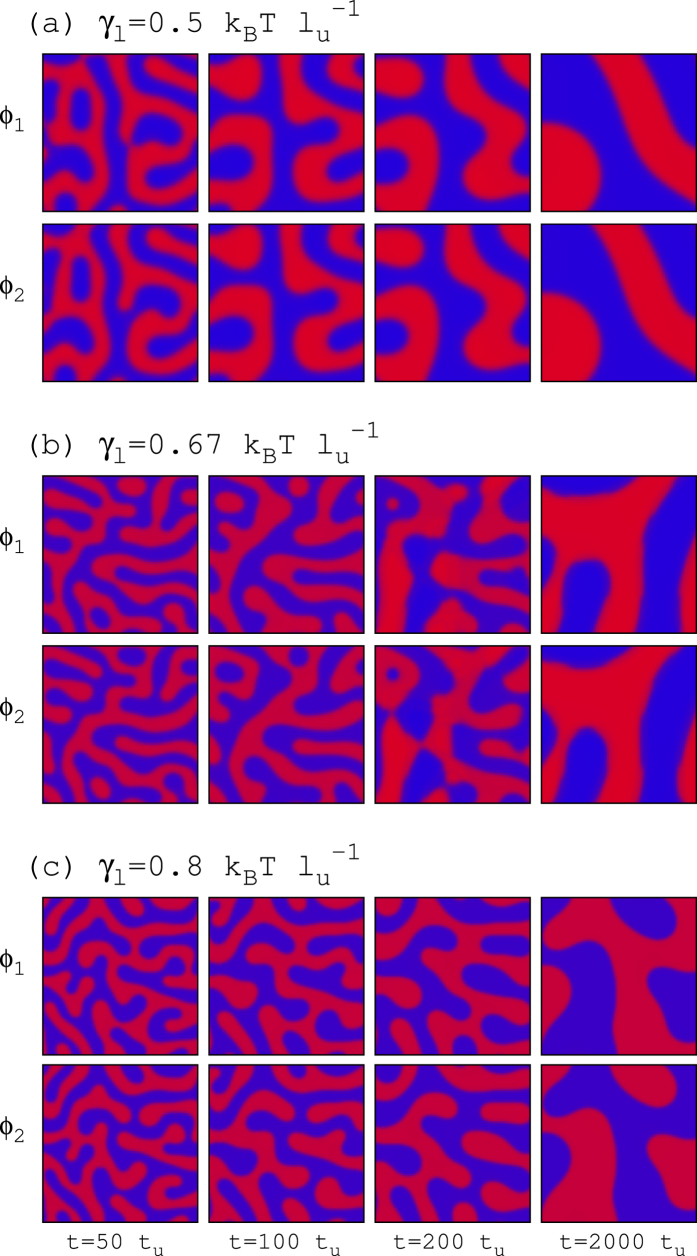
Evolution of registered and antiregistered segregation patterns in unperturbed bilayers. Temporal sequence of the lipid composition at both layers obtained from simulations of pure lipid bilayer systems (*λ* = 0) for *γ*_*s*_ = 0.4 and different values of *γ*_*l*_. Blue and red colors stand for *ϕ*^(*Ld*)^ and *ϕ*^(*Lo*)^ segregating phases, respectively. The linear size of the system is 100 *l*_*u*_.

**Figure 7:**
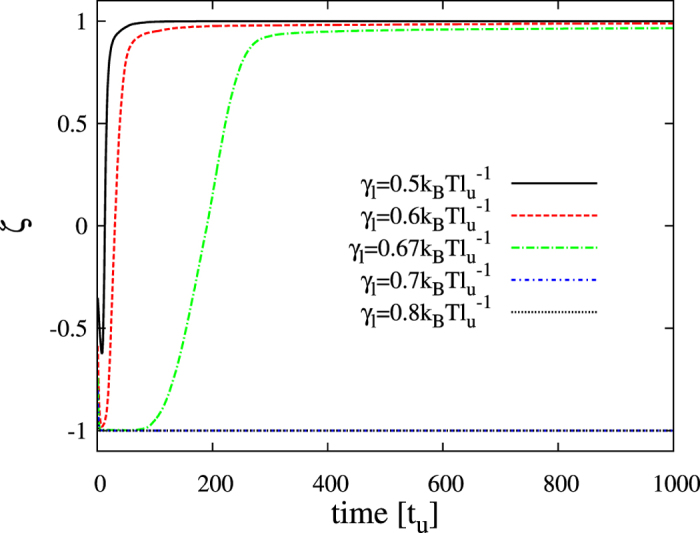
Evolution of the transversal correlation in unperturbed segregating bilayers. Temporal behavior of the correlation variable *ζ* obtained from simulations of pure lipid bilayer systems (*λ* = 0) for *γ*_*s*_ = 0.4 and different values of *γ*_*l*_.

The differential kinetic equations (8) and (10) are numerically solved (see Methods) for *α* = 2, *γ* = 1 and *β* = 1up to *t* = 1000*t*_*u*_. For unperturbed bilayers (*λ* = 0) the simulation results are summarized in Figs 6 and 7. In the registration region of the phase diagram (*γ*_*s*_ = 0.4 and *γ*_*l*_ = 0.5 in Fig. 4a), growing stripe patterns develop until complete spinodal decomposition (Fig. 6a). Although at the very initial segregation stages (not seen in Fig. 6a) the emerging composition modulations display transversal anticorrelation, phase registration is rapidly exhibited. In order to quantify the degree of the transversal phase symmetry of the segregating domains and its temporal evolution, I define the parameter


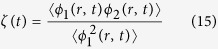


where the brackets stand for spatial average. This sort of correlation parameter quantifies the transversal alignment of lipid domains from *ζ* = −1 (full anticorrelation) to *ζ* = +1 (complete coincidence). The duration of the initial anticorrelated transient observed for the case *γ*_*l*_ = 0.5 is increased for larger thickness mismatch tensions (see Fig. 7). For instance, the pattern evolution for the case *γ*_*l*_ = 0.67 has been plotted in Fig. 6b and clearly exhibits phase asymmetry the first two snapshots before changing and adopting complete domain alignment (see also Fig. 7 for the quantification of this effect). A little increment of thickness line tension results in a permanent antiregistered segregation behavior for the whole simulation period. For these limiting cases, twice-longer simulations have been run and the antiregsitration behavior persists. Much larger values of *γ*_*l*_ also display robust and persistent phase asymmetry (see Figs 6c and 7).

These results qualitatively agree with the predictions obtained from the linear stability analysis (Fig. 4). However, the nonlinear terms in Eqs (8) and (10), which were discarded in the linear analysis, favor the transition from antiregistered to registered modes in the simulations, providing boundary values for 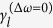 larger than those predicted in equation (13). The new boundary, 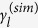, can be computed by performing series of simulations at progressively increasing values of *γ*_*l*_. Its value is then assigned as the smallest *γ*_*l*_ that leads to *ζ*(1000*t*_*u*_) > 0. 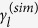 has been computed at different values of *γ*_*s*_ by using small increments of 0.002 for the variable *γ*_*l*_, and performing statistics over 5 different initial configurations of the simulated systems. The results are shown in Fig. 4a for unperturbed membranes, and I checked that twice-longer simulations do not modify the computed boundary curve. Above 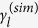, simulations exhibit permanent phase asymmetry for the whole simulation period. Notice that in these cases, the antiregistered configuration does not correspond to equilibrium but to a metastable conformation. In this study, fluctuations in local lipid composition have not been taken into account, and they could be considered by introducing appropriate noise terms in the kinetic equations. Due to its effect, metastable anticorrelated states may evolve to the transversal alignment expected for large segregated domains at equilibrium. As a consequence, the values for the boundary line tension parameter 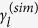 may eventually increase. A complete analysis of the metastability of the antiregistered modes, however, is beyond the scope of this Paper.

**Figure 8:**
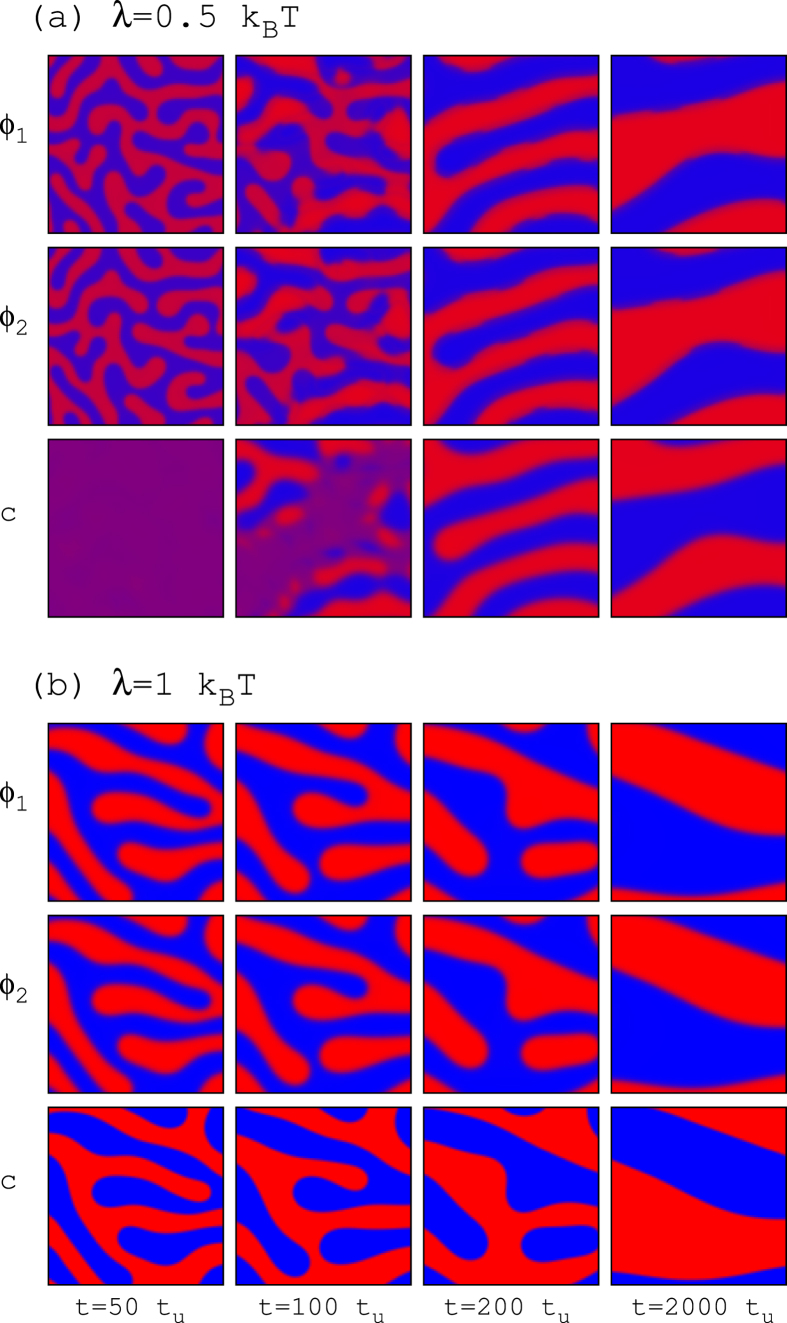
Evolution of registered and antiregistered segregation patterns in the presence of a fast translocating compound. Temporal sequence of the compositional parameters obtained from simulations for *γ*_*s*_ = 0.4, *γ*_*l*_ = 0.8, *D*_*c*_ = 10 and different values of the affinity parameter *λ*. The color scale for lipids and the size of the system are the same as in Fig. 6. Positive (negative) deviations for the distribution of *c(r*) are plotted in red (blue).

**Figure 9:**
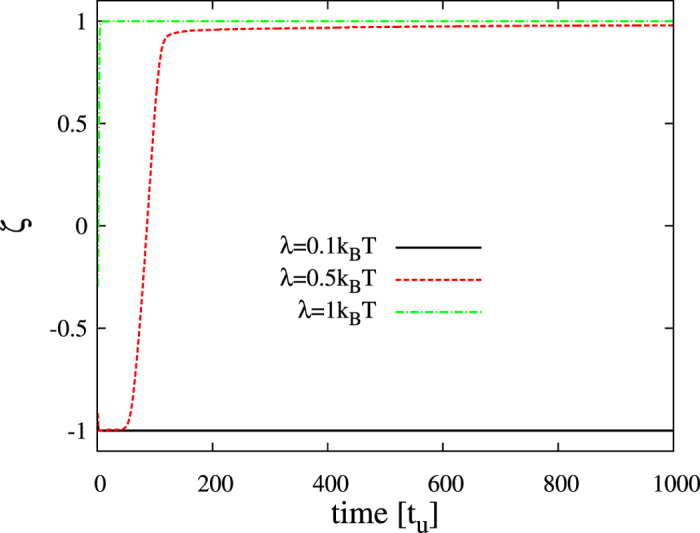
Evolution of the transversal correlation in the presence of a fast translocating compound. Temporal behavior of the correlation variable *ζ* obtained from simulations for *γ*_*s*_ = 0.4, *γ*_*l*_ = 0.8, *D*_*c*_ = 10 and different values of the affinity parameter *λ*.

The effects due to a rapidly translocating compound are clearly visualized in Fig. 8 for the case *γ*_*s*_ = 0.4 and *γ*_*l*_ = 0.8 that displays phase asymmetry in unperturbed conditions (Fig. 6a). Addition of the non-lipid compound with only slight preferences for one of the two lipid phases (small *λ*) does not change this behavior. Above a certain value, however, the initial anticorrelated configuration evolves to complete phase alignment simultaneously with domain coarsening. Figure 8a shows the case *λ* = 0.5: the first snapshot displays antiregistration and absence of modulation in the distribution of the non-lipid compound. At longer times (second snapshot in Fig. 8a) a pattern of modulated stripes for the variable *c(r*) arises, forcing the alignment of the two lipid compositional parameters (third and fourth panels in Fig. 8a). This transition is confirmed by the temporal evolution of the correlation variable *ζ* (Fig. 9). Larger preference for a lipid phase shortens the anticorrelated transient and results in complete registration from the very initial stages of the simulation (see for example the case *λ* = 1 in Figs 8b and 9).

**Figure 10:**
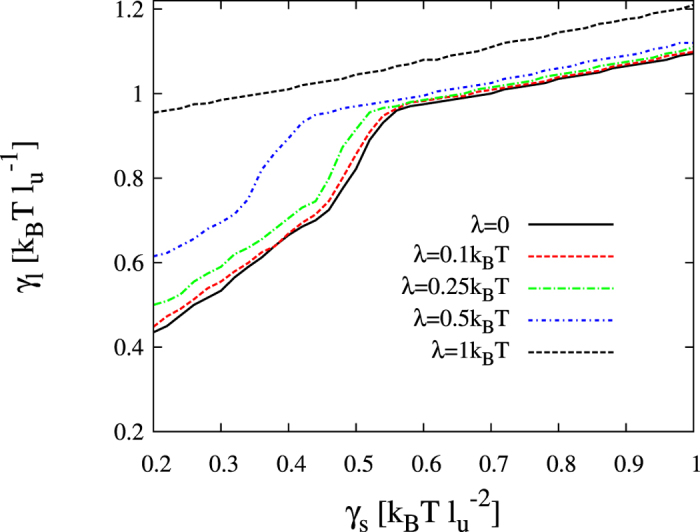
Kinetic phase diagram under the effect of a fast translocating compound. Modification of the kinetic stability diagram boundary 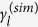 for different values of the affinity parameter *λ*. The region below (above) the boundary corresponds to the registration (antiregistration) regime in the numerical simulations.

Following the same procedure described above, the boundary obtained from numerical simulations, 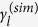, has been computed in the presence of a fast translocating compound. For the largest affinity parameter, *λ* = 1, almost the totality of the (*γ*_*s*_, *γ*_*l*_)-parameter space plotted in Fig. 5a corresponds to the registration region. A systematic analysis is provided in Fig. 10 for different values of the affinity parameter *λ*. For compounds having a weak preference for a lipid phase, only a tiny modification of the phase diagram boundary is observed, whereas compounds exhibiting a strong affinity to be placed in one of the lipid phases cause a significant expansion of the registration region of the phase diagram (Fig. 10). Both aspects (rapid translocation behavior and strong preference for a particular lipid phase) are therefore required to modify interleaflet coupling in phase segregating membranes.

## Conclusions

By using different modeling and numerical perspectives I have analyzed the effects caused by specific compounds when added to a phase segregating lipid bilayer system. More specifically, I address compounds that are preferentially placed in specific lipid environments and exhibit a fast exchange between the layers of the membrane. In particular, a collection of CG MD simulations of compositionally symmetric phase separating bilayers has analyzed the effects of chloroform on the transversal alignment of segregating domains. The simulations show a clear alteration of the membrane interleaflet correlation by promoting domain registration upon addition of chloroform. The analysis of the lateral and transversal behavior of chloroform molecules at the molecular detail unveils the entropic origin of the reported effect: transversal colocalization of similar lipid phases favors the rapid exchange of chloroform between the layers of the membrane avoiding entropically disfavored confinement in one of the leaflets. Negative control MD simulations using non-translocating compounds confirm this rationale. This hypothesis has been also tested by means of a continuum, free energy based, approach. Following a simple scheme, the transversal behavior of a segregating bilayer is determined by the competition between a surface interleaflet tension and line thickness mismatch tension. When perturbed by a compound that displays a preference for one of the two segregating lipid phases, two extreme scenarios can be compared depending on whether the added compound is allowed to rapidly jump from one layer to the other or not. In the former case, there is an energetic gain by aligning the segregating domains at both sides of the membrane, respect to the local confinement of the added compound to one of the two layers. The kinetic equations derived from the free energy derivation double-check this result: both the linear stability analysis and numerical resolution of the kinetic equations reveal that the addition of a fast translocating compound favors transversal correlation of segregating domains.

A simple quantitative estimation of the entropic effect due to inclusion of chloroform (or any other rapidly translocating compound) can be performed. A rough (upper) estimation of this effect is obtained by assuming that chloroform molecules are much more mobile in the transversal direction than in the membrane plane (lipid molecules being placed in a ‘vertical’ position, reduce the lateral mobility of chlf). In the limit of ideal particles moving only in the ‘vertical’ direction, the variation of entropy due to the confinement of N chlf particles from a system of length L to L/2 corresponds to 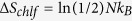; namely, to a positive contribution to the free energy, 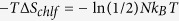 that penalizes transversal domain antiregistration. The magnitude of this entropic contribution is found to be relevant with respect to the two competing line and surface tension terms discussed along this Paper. For instance, the magnitude of the interleaflet surface tension (≈0.1 k_B_T/nm^2^) is equivalent to the previous entropic penalty for a chloroform surface concentration of about 0.14 molecules/nm^2^. Notice that the reported MD simulations contain about 3 chloroform molecules per nm^2^ of membrane, so that the suggested entropic effect can be clearly captured.

The reported transversal synchronizing effect can be generalized to all compounds displaying a preference for any of the segregating lipid phases and a high exchange frequency between layers. This is normally accomplished by small amphipathic molecules exhibiting a slight polar affinity, which causes this compound to partition inside the bilayer with a preference to interact with the lipid/water interface at both leaflets, and therefore to periodically jump from one layer to the other. Such partitioning behavior has been also found in MD simulations for other compounds than chloroform like ketamine^40^, halothane^41^ or xenon^42^. Interestingly all these compounds are endowed with anesthetic effects, indicating that the reported synchronizing effect may be of importance to understand the anesthetic action. Actually, the main difference between many anesthetics and similar non-anesthetic counterparts is that the former localizes close to the lipid/water interface according to experiments^43^. For example, the non-anesthetic carbon tetrachloride, similar to chloroform but lacking in polar affinity, is preferentially located at hydrophobic midplane region of the membrane^32^,^33^. As a consequence, its addition to a phase segregating membrane did not imply a significant leaflet coupling effect^29^. A comparative MD study performed for various noble gases^44^ reveals that xenon (the one with the strongest narcotic efficiency) displays the largest probability to be placed in the interfacial region between lipid tails and headgroups. Compounds with a strong polar character, for instance ethanol^45^ or dimethyl sulfoxide^46^, are more prone to occupy the lipid/water interfacial regions but avoiding the inner hydrophobic bilayer core. When added to a segregating membrane system, their molecules rarely traverse the hydrophobic section of the bilayer, and consequently, no registration effect is expected^29^.

In a more general context beyond the anesthetic action, many other relevant biological functions taking place in the cell membrane, like transmembrane signaling or the activity of many ion channels and transmembrane proteins, require a particular interleaflet correlation. How the presence of specific compounds may alter the phase cross-talk between the two leaflets of the membranes is therefore of key importance to understand the effects of these compounds on the biological function. So far, the lateral organization of the cell membrane has been conjectured to play a role in the cell membrane functionality. The findings presented here contribute to the latter discussion by introducing the possibility that not only in-plane but also transversal modifications of the membrane organization have to be taken into account.

## Methods

### Molecular dynamics simulations: details and protocols

The simulations were performed using the GROMACS v.4.5.5 software package^47^. The Martini v2.0 force field provided the CG description of the simulated molecules by lumping together an average of 4 atom groups on a single bead, except for cholesterol, whose ring-like structure is mapped with a higher 3-to-1 resolution^48^. The Martini model has been successfully applied to study membrane domain formation and phase behavior (see ref. 49 and references therein). The simulations were carried out in the NpT ensemble through a weak coupling algorithm at T = 295 K and an anisotropic p = 1 atm. Electrostatic interactions were handled using a shifted Coulombic potential energy form and charges were screened with a relative dielectric constant ε_r_ = 15. Periodic boundary conditions were used in all three directions, and the time step was set to 20 fs. I used the standard conversion factor of 4 which is the speed-up factor needed to obtain the correct diffusional dynamics of CG water particles compared with real water molecules^48^. All simulated membranes had an approximate surface of 500 nm^2^ and displayed Lo/Ld coexistence.

Several checks were performed by conducting simulation tests with different protocols and conditions^29^. I checked the use of different coupling schemes for temperature and pressure, and we found that the main reported results were not significantly altered by the use of Nosé-Hoover thermostat and Parrinello-Rahman barostat instead of Berendsen weak coupling approaches. The use of polarizable water^50^ did not modify the simulations outcome either. Although the size of the simulated membranes (about 500 nm^2^) is standard in many of the most recent simulations using CG simulations^49^ caution has to be exercised when membrane domains become of the order of the system size and periodic boundary conditions are used (the so called finite size artifacts). The simulation of a 2 × 2 replica of the initially mixed DUPC/DSPC/chol system has discarded possible finite size effects. These enlarged membranes contained 7776 lipid molecules and 50400 water particles, summing up a total of 138816 beads, and its lateral size was of the order of 45 nm (a surface of about 2000 nm^2^). This membrane was simulated both in the absence of chloroform and in the presence of 6000 chlf molecules (the same ratio of 0.77 chlf per lipid used in the original membrane) up to 4,8 μs. Visual inspection of the evolution of the enlarged membranes revealed a consistent effect of chloroform on the transversal behavior of segregating domains.

Finally, since biomembranes are generally immersed in an aqueous medium with a high ionic strength, I also checked the effect of an electrolyte in our results. DUPC/DSPC/chol membrane system was simulated (with and without chlf) in the presence of Na^+^ and Cl^−^ 0.15 M (similar to the typical physiological ionic strength). The effect of chloroform on transversal domain synchronization was fully reproduced in these simulations in accordance to the results in the absence of electrolyte.

### Structural analysis of CG simulated membranes

Structural analysis of the simulated membranes was performed by the use of Voronoi tessellation. For a given leaflet, phosphate beads of PC molecules and hydroxyl groups of chol molecules were projected onto a plane. The Voronoi tessellation was performed for each leaflet according to the mentioned projections. Because each Voronoi polygon is associated with an individual molecule, membrane properties can be computed and differential values can be assigned to each component of the membrane.

Quantification of lateral phase segregation and transversal phase registration are performed as follows. DUPC molecules were considered to form the Ld phase, whereas the saturated PC species corresponded to the Lo phase. Cholesterol molecules were considered to be part of the Lo phase except in the cases where they had more DUPC neighbors than saturated and other chol molecules. According to the Voronoi tessellation, for each lipid molecule several lateral (in-plane) neighbors and a unique transmembrane neighbor can be identified. The phase segregation process can be tracked by computing the proportion of lateral neighboring pairs that correspond to lipid molecules of the same phase. Composition, leaflet thickness and lateral mobility have been therefore computed for each lipid phase using the last 4 μs of simulation. On what respects to the transversal behavior, for a given lipid in one leaflet, the area of its Voronoi polygon is considered to be registered if its transversal neighbor corresponds to the same lipid phase. The global registration percentage can be then computed by averaging all pairs of transmembrane neighbor lipids.

### Numerical resolution of the kinetic equations

The differential kinetic equations (8) and (10) were numerically solved in a two-dimensional lattice of 100 × 100 sites for the compositional order parameters *ϕ*_1_, *ϕ*_2_ and *c*. Periodic boundary conditions were applied. The discretization mesh was chosen Δ*x* = 1*l*_*u*_, spatial derivatives were calculated employing a simple centered scheme, and a first order Euler algorithm with time step Δ*t* = 0.001*t*_*u*_ was used for temporal integration. Both choices assured a good numerical convergence. Simulations were started from a homogeneous distribution *ϕ*_1_(*r*) = *ϕ*_2_(*r*) = *c(r*) = 0, slightly perturbed with local variations of ±1%.

## Additional Information

**How to cite this article**: Reigada, R. Alteration of interleaflet coupling due to compounds displaying rapid translocation in lipid membranes. *Sci. Rep.* **6**, 32934; doi: 10.1038/srep32934 (2016).

## Figures and Tables

**Figure 1:**
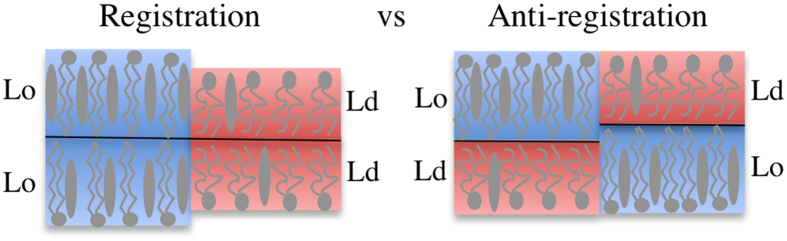
Schematic representation of phase registration and antiregistration modes. Ternary lipid membranes may segregate in ordered (Lo, in blue) domains rich in saturated lipids (elongated and grey) and cholesterol (grey disks) and disordered (Ld, in red) domains rich in unsaturated lipids (short and grey). Two configurations can be then displayed: phase registration (left) is achieved when the energy penalty due to the thickness mismatch is lower than the interleaflet tension at the interface of distinct lipid phases, whereas antiregistration (right) appears in the opposite case.
